# Data of small peptides in SMILES and three-dimensional formats for virtual screening campaigns

**DOI:** 10.1016/j.dib.2019.104607

**Published:** 2019-10-04

**Authors:** Vivitri Dewi Prasasty, Enade Perdana Istyastono

**Affiliations:** a Faculty of Biotechnology, Atma Jaya Catholic University of Indonesia, Jakarta, 12930, Indonesia; b Faculty of Pharmacy, Sanata Dharma University, Paingan, Maguwoharjo, Sleman, Depok, Yogyakarta, 55282, Indonesia

**Keywords:** Small peptide, Drug discovery, Virtual screening, Dipeptide, Tripeptide, Tetrapeptide

## Abstract

The data presented in this article are structures of dipeptides, tripeptides and tetrapeptides constructed from all possible combinations of 20 natural and common amino acids. In total, the data contains 168400 peptides. The structures are available in their simplified molecular-input line-entry system (SMILES) and three-dimensional (3D) formats. The type of data are text files, which could be accessed and modified either by text editor applications (*e.g.* Notepad++) or by molecule visualization softwares (*e.g.*, YASARA View). These structures could be used further in virtual screening campaigns in the early stage of drug discovery projects.

**Table:** Specifications Table

Subject area	*Pharmaceutical Sciences*
More specific subject area	*Drug discovery*
Type of data	*Text file*
How data was acquired	*Molecular modelling*
Data format	*Raw*
Experimental factors	*The data were produced by employing a computer server with Ubuntu Linux 14.04.5 as the operating system, 8GB RAM and 8 virtual CPUs of 6.4 GHz*
Experimental features	*The data were built by employing two main computational chemistry tools, i.e., molconvert 17.13.0 from ChemAxon and gen3d from Open Babel version 2.3.0.*
Data source location	*Data were built using molecular modelling techniques in a virtual laboratory owned by Sanata Dharma University, Yogyakarta, Indonesia.*
Data accessibility	*Mendeley Data.* doi:http://dx.doi.org/10.17632/z8zh5rpthg.1
Related research article	*Prasasty, V. Radifar, M., Istyastono, E., 2018. Natural peptides in drug discovery targeting acetylcholinesterase. Molecules. 23(9): 2344.*https://doi.org/10.3390/molecules23092344 [1]

**Table:** 

**Value of the Data**
• The data provide structures of all possible natural dipeptides, tripeptides and tetrapeptides to be employed further in virtual screening campaigns.
• The SMILES formats of the peptides provide opportunities to perform virtual screening campaigns from the very beginning starting point.
• The 3D structures in mol2 formats provide opportunities to perform virtual screening campaigns from the 3D structure as the starting point.
• Since most of the peptides have molecular weight of less than 500 Da, the resulted hits in the virtual screening campaigns could be optimized further to be potential ligands in drug discovery projects.

## 1 Data

**Fig. 1:**
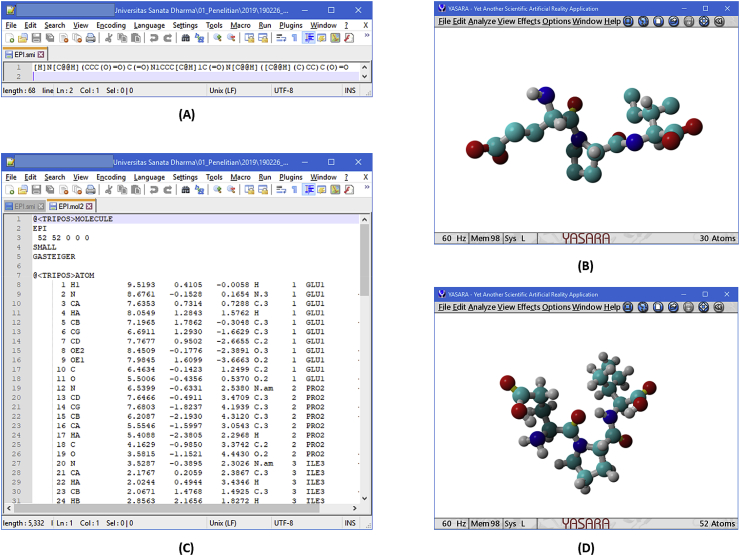
The EPI.smi in Notepad++ (A) and YASARA View (B), and the EPI.mol2 in Notepad++ (C) and YASARA View (D).

There are three files in the data: dipeptides.zip, tripeptides.zip, and tetrapeptides.zip. Each file contains two subdirectories, *i.e.*, 3d-in-mol2 and smi. The directories 3d-in-mol2 and smi contain structures in 3D in mol2 format and structures in SMILES format, respectively. For dipeptides.zip and tripeptides, those directories contain structure files of the peptides named by their sequences in one-letter-code. These dipeptides and tripeptides have been subjected in a structure-based virtual screening to discover novel acetylcholinesterase (AChE) inhibitors [1] by employing a retrospectively validated protocol [2]. In tetrateptides.zip, the 3d-in-mol2 and smi directories contain subdirectories named with the first sequence of the peptides inside the directory followed by triple x. For example, Fig. 1 shows tripeptide EPI in the smi and the mol2 formats visualized using both Notepad++ as a text editor and Yasara View as a molecule visualization application. The file EPI.smi could be found in the subdirectory smi, while the file EPI.mol2 could be found in the subdirectory 3d-in-mol2 in tripeptides.zip.

## 2 Experimental design, materials, and methods

The data were built in a computer server with Ubuntu Linux 14.04.5 as the operating system, 8GB RAM and 8 virtual CPUs of 6.4 GHz. First of all, three input files named dipeptides.lst, tripeptides.lst, and tetrapeptides.lst were made. These files contained all possible sequences of dipeptides, tripeptides, and tetrapeptides in their one-letter-code, respectively. Subsequently, the smi files were built by using module *molconvert* (Molecule File Converter, version 17.13.0) from ChemAxon (https://chemaxon.com/). Based on these smi files, the 3D structures were then built using module *gen3d* from Open Babel version 2.3.0 [3]. The *molconvert* module requires amino acids sequence to be converted to SMILES format. The following is, for example, the generic code to build the dipeptides presented in this article: *for i in $(cat dipeptides.lst); do mkdir $i; cd $i; molconvert –peptide $i smiles* > *$i.smi; babel –title $i –gen3d -ismi $i.smi -omol2 $i.mol2; cd ..; done*.

The availability of the data will significantly reduce time to perform virtual screening campaigns. The following are some examples of study that could benefit from the data: (i) PeptoGrid - Rescoring function for AutoDock Vina to identify new bioactive molecules from short peptide libraries [4], (ii) Structure-based virtual screening for fragment-like ligands of the G protein-coupled histamine H4 receptor [5], and (iii) Natural peptides in drug discovery targeting acetylcholinesterase [1].
